# Elemental mercury poisoning caused by subcutaneous and intravenous injection: An unusual self-injury

**DOI:** 10.4103/0971-3026.63056

**Published:** 2010-05

**Authors:** Jaywant Wale, Pankaj K Yadav, Shairy Garg

**Affiliations:** Department of Radiodiagnosis, G.R. Medical College, Gwalior, India

**Keywords:** Intravenous, mercury, subcutaneous

## Abstract

Elemental mercury poisoning most commonly occurs through vapor inhalation as mercury is well absorbed through the lungs. Administering subcutaneous and intravenous elemental mercury is very uncommon but with only a few isolated case reports in the literature. We present an unusual case of elemental mercury poisoning in a 20-year-old young male who presented with chest pain, fever, and hemoptysis. He had injected himself subcutaneously with elemental mercury obtained from a sphygmomanometer. The typical radiographic findings in the chest, forearm, and abdomen are discussed, with a review of the literature.

## Introduction

Mercury (Hg)[1] is the only metal that is liquid and easily vaporized at room temperature. It occurs in three forms: elemental mercury, inorganic mercury salts, and organic mercurial compounds. Mercury poisoning can result from inhalation of the vapor, ingestion, injection, or absorption through the skin.

Elemental mercury poisoning results most commonly because of vapor inhalation, as it is absorbed (80%) throughout the lungs.[2] Ingestion of elemental mercury causes virtually no toxicity as it has poor gastrointestinal absorption.[1] Poisoning due to intravenous injection of mercury usually occurs in connection with attempted suicide, by accident, or in drug addicts exploring new ways to become intoxicated.[3] Subcutaneous and intravenous injection of elemental mercury is very uncommon, with only isolated case reports in the literature.

## Case Report

A 20-year-old man presented with diffuse chest pain for three days and moderate grade fever for one day with hemoptysis. About three months earlier he had injected approximately 5 ml of elemental mercury subcutaneously into his left forearm; in addition, seven days prior to presentation he had injected approximately 8-10 ml of elemental mercury (obtained from a sphygmomanometer) intravenously.

On examination, the patient was conscious, oriented, and afebrile. Puncture marks were seen in the left antecubital fossa. Abscess formation at the injection site and small amount of mercury could be seen coming out from the puncture sites. The blood urea, serum creatinine, and urine analysis were normal.

**Figure 1:**
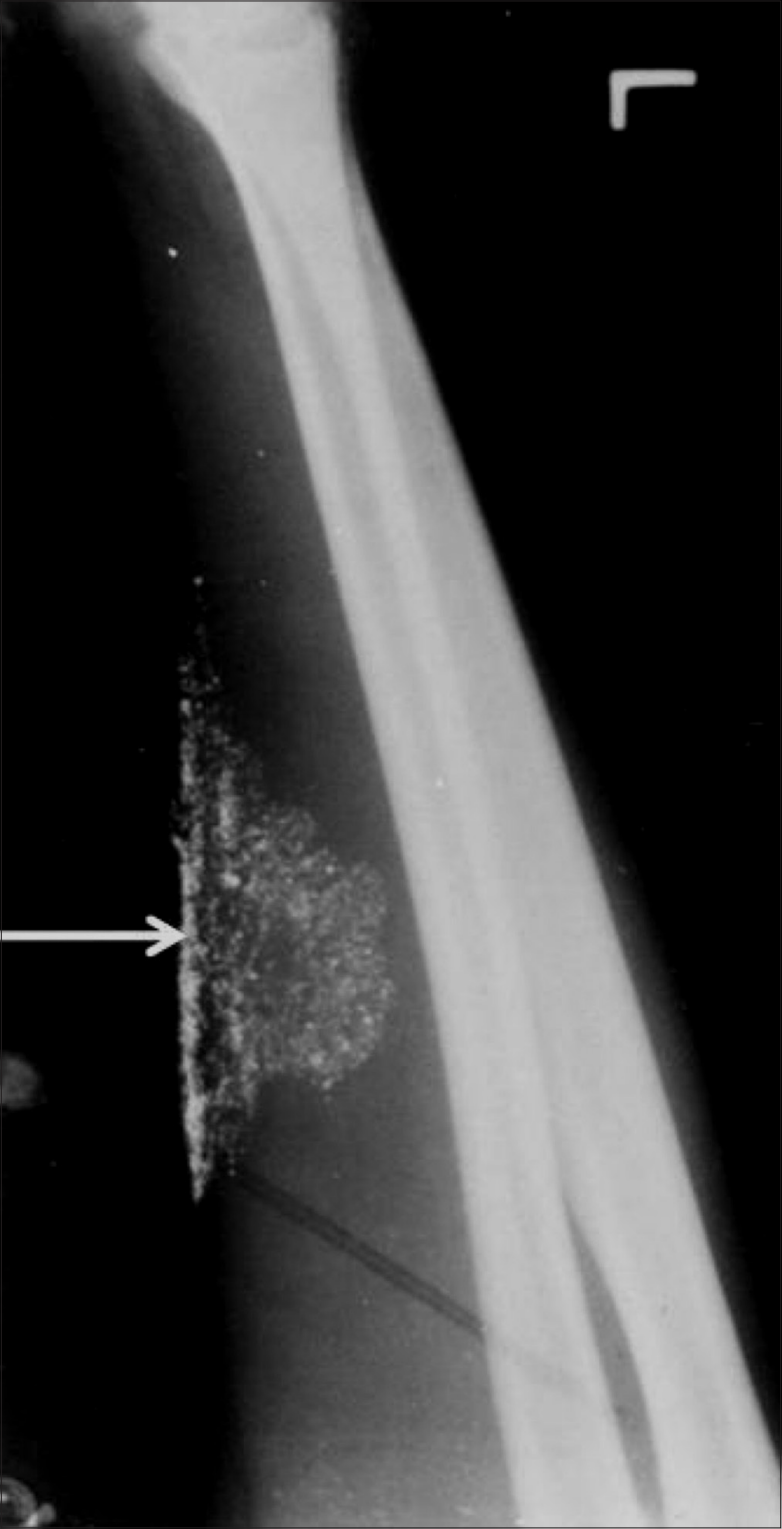
Oblique radiograph of the left forearm shows fine nodular opacities at the site of injection (arrow)

**Figure 2:**
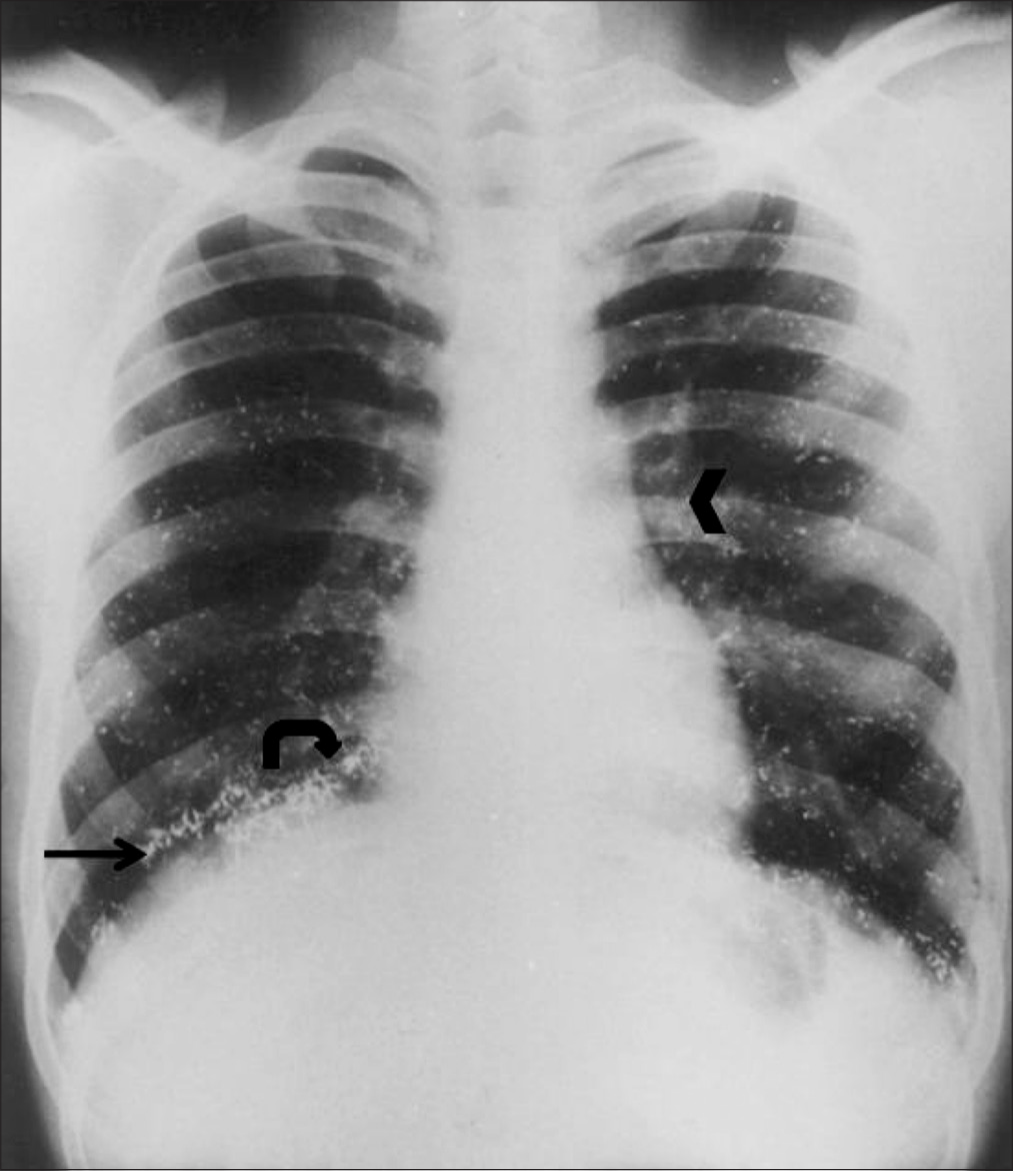
Radiograph of the chest shows multiple tiny metallic densities arranged in a discrete fashion throughout the lung parenchyma bilaterally, predominantly at the lung bases (arrow). Densities are also seen in both hila (arrowhead) and at the cardiophrenic angles (curve arrow)

A plain radiograph of the left forearm revealed multiple tiny metallic densities in the soft tissues at the site of the subcutaneous injection [Figure 1]. A plain radiograph of the chest revealed multiple tiny, discrete, metallic densities throughout the lung parenchyma bilaterally, predominantly at the lung bases. The cardiac shadow was normal. Similar metallic densities were also seen at both pulmonary hila and at both cardiophrenic angles [Figure 2]. A lateral radiograph of the skull was found to be normal.

**Figure 3:**
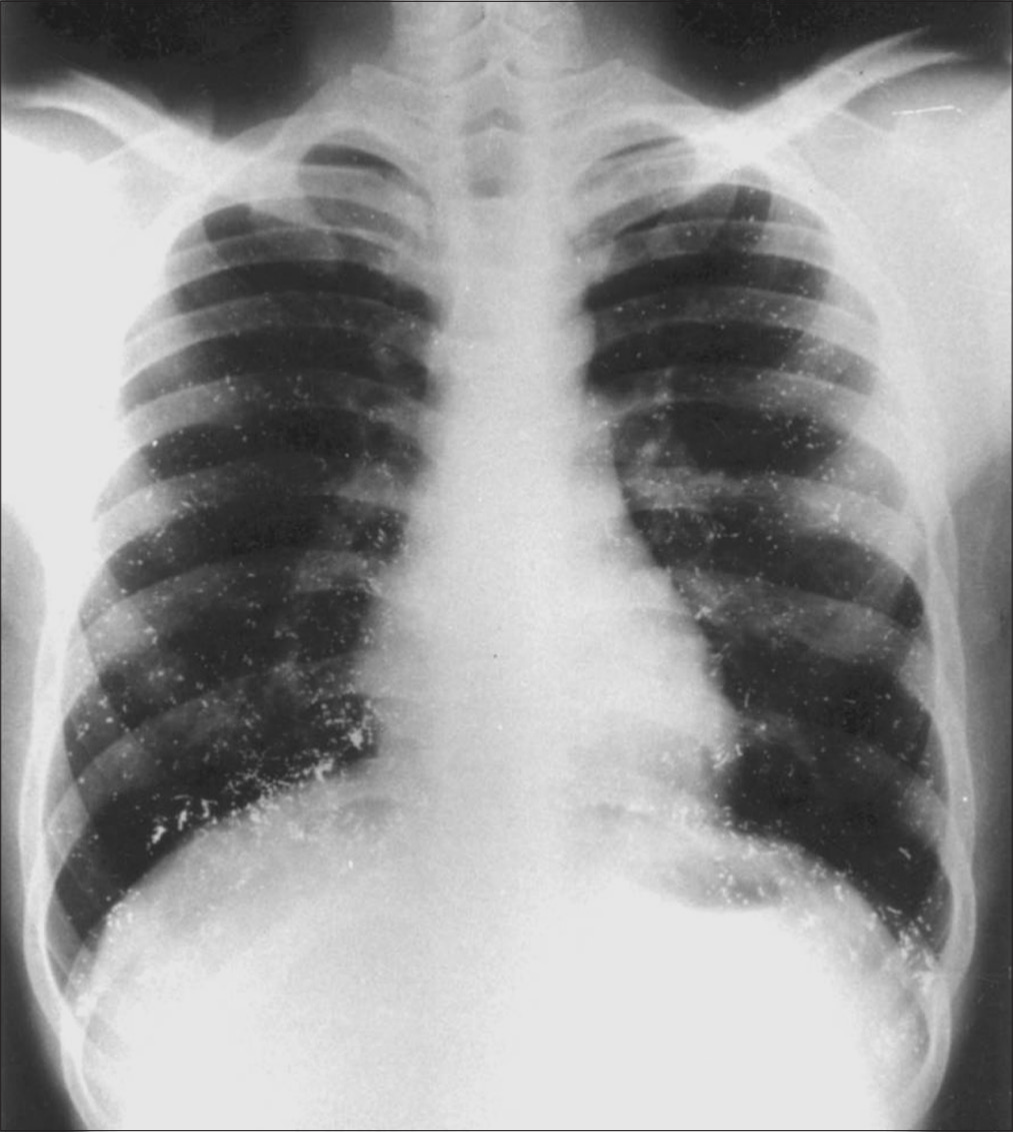
Follow-up radiograph of the chest after 6 weeks shows minimal regression of findings

**Figure 4:**
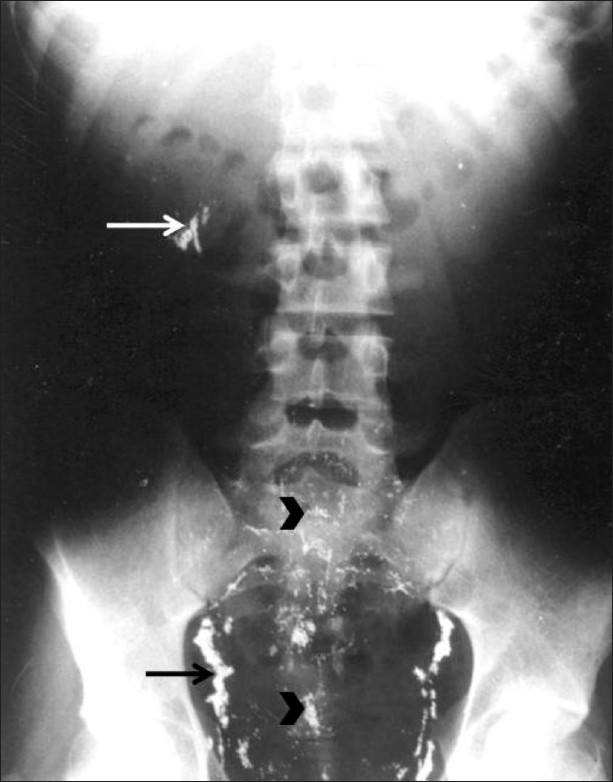
Follow-up radiograph of the abdomen shows tiny metallic densities in the region of the ureter and the pelvicalyceal system (arrows). Densities are also seen over the lower lumbar spine and the pelvis (arrowheads)

The patient was prescribed the chelating agent penicillamine for a few months, along with erythromycin for 10 days. A follow-up chest radiograph obtained at six weeks showed some diminution in the quantity of mercury deposits in the lungs [Figure 3]. A plain radiograph of the abdomen revealed multiple tiny metallic densities in the region of the pelvicalyceal systems and both lower ureters. These densities were also seen over the lower lumbar spine and in the pelvis, arranged in a thin linear pattern [Figure 4]. A radiograph of the forearm revealed no significant change from the initial picture.

## Discussion

Mercury is the only common metal that is in liquid form at room temperature. It is used in making thermometers, manometers, electronic devices, mercury vapor lamps, skin ointments, dental amalgams. Due to a high water-metal interfacial tension and lack of bonding to other materials, mercury takes the form of tiny spherules or coalesces when it enters the plasma. Pulmonary embolization is rare and when it does occur it is either accidental or it is due to deliberate self-injection by people, either for self-intoxication or for attempting suicide.[4] The mercury which is disseminated throughout the pulmonary circulation by the right ventricle, gives rise to a distinctive radiological appearance due to its high atomic weight. Micro nodular or tubular opacities may be present, depending mainly on the dose of mercury exposure; these opacities are distributed symmetrically and bilaterally, as seen in our patient. As mercury may pass through the pulmonary capillaries and precapillary shunts it can enter the systemic circulation, as seen in our patient, with metallic densities noted over the renal and ureteric areas. The metallic densities over the lumbar spine and pelvis were arranged in a thin linear pattern, and were probably in the capillaries.

In cases of intravenous self-administration in the forearm veins, aggregations of mercury at the site of injection may be radiologically seen.[5]

Mercury is toxic in all forms. Elemental mercury, when injected intravenously, can cause widely varying presentations: from total lack of symptoms (the diagnosis being made incidentally) to respiratory failure, kidney damage, liver damage, neurologic symptoms, and even death. The toxic effects of mercury after subcutaneous injection are not as serious as that seen after acute inhalation. Nevertheless, once mercury enters the bloodstream, it is quickly distributed throughout the body, particularly in the lungs.[6]

In cases of mercury poisoning, close monitoring of the clinical status of the patient is required with follow-up for at least two years.[6] Many case reports have described similar findings as in our patient.[7–9] Mercury is mainly excreted from the body by the kidneys, but the rate of excretion is usually very slow and traces of mercury can be seen on radiographs and in urine even two years after the index event.[6] In our case too, radiographs taken after six weeks did not show any significant change. Our patient, however, made a full clinical recovery and did not show any renal, hepatic, neurologic, or pulmonary sequelae over two years of follow-up.
